# Prevalence and metabolic determinants of abnormal alanine aminotransferase: A cross‐sectional study of Iranian adults, 2018–2022

**DOI:** 10.1002/jcla.24937

**Published:** 2023-07-05

**Authors:** Samaneh Asgari, Danial Molavizadeh, Maryam Tohidi, Amir Abbas Momenan, Fereidoun Azizi, Farzad Hadaegh

**Affiliations:** ^1^ Prevention of Metabolic Disorders Research Center, Research Institute for Endocrine Sciences Shahid Beheshti University of Medical Sciences Tehran Iran; ^2^ School of Medicine Kashan University of Medical Sciences Kashan Iran; ^3^ Endocrine Research Center, Research Institute for Endocrine Sciences Shahid Beheshti University of Medical Sciences Tehran Iran

**Keywords:** alanine aminotransferase, prevalence, metabolic determinants, logistic regression

## Abstract

**Background:**

Alanine aminotransferase (ALT) is an enzyme whose activity became the principal biomarker for liver disease. In the current study, we aimed to determine the prevalence of abnormal ALT, as a surrogate of nonalcoholic fatty liver disease (NAFLD) and its associated determinants using different criteria among Tehranian subjects between 2018 and 2022.

**Methods:**

This is a cross‐sectional study on 5676 Tehranian individuals aged 20–70 years. The weighted prevalence of abnormal ALT was calculated using both the National Health and Nutrition Examination Survey in the United States (US‐NHANCE; ALT ≥30 U/L for females and ≥40 U/L for males) and the American College of Gastroenterology (ACG) guideline (ALT >25 U/L for females, and >33 U/L for males) thresholds. Moreover, uni/multivariable logistic regression analysis was performed to find the determinants of abnormal ALT.

**Results:**

The weighted prevalence of abnormal ALT was 12.8% (7.6% females and 18% males) and 22.5% (17.7% females and 27.3% males) based on US‐NHANCE and ACG criteria, respectively. Our results showed every decade increase in age decreased the risk of abnormal ALT by 32%. We also found that generally male gender, being overweight/obese, central adiposity, TG ≥6.9 mmol/L, non‐HDL‐C ≥3.37 mmol/L, lipid‐lowering medications, pre‐diabetes/T2DM were associated with abnormal ALT using different cutoff points. Moreover, among men resting tachycardia (≥90 beats per min), hypertension, and females past‐smoker were also found as other determinants of abnormal ALT.

**Conclusion:**

High prevalence of abnormal ALT among non‐elderly Iranian adults, especially among men, necessitates immediate multifaceted strategies by policymakers to prevent potential complications caused by NAFLD.

## Graphical abstract

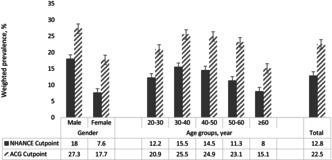

## 1 INTRODUCTION

Alanine aminotransferase (ALT) is an enzyme that principally presents in the cytosol of the hepatocytes with more than three thousand activities compared to its serum activity. ALT also exists in other tissues including, the kidney, heart, pancreas, muscles, adipose tissues, intestines, colon, prostate, and brain with a much lower concentration than the liver.^1^ Among different liver enzymes, serum ALT activity became the principal biomarker for liver disease^2^ such as viral hepatitis, autoimmune hepatitis, and most importantly nonalcoholic fatty liver disease (NAFLD).^3^, ^4^

Based on the data from the National Health and Nutrition Examination Survey (NHANES), the prevalence of elevated ALT raised from 8.9% in 2002 (ALT >43 IU/L for both genders) to 11.4% in 2012 (>40 IU/L for males, >31 IU/L for females) in the U.S adults.^5^, ^6^ Also, there is a similar growing trend for the prevalence of elevated ALT in the Korean population; from 7.4% (>43 U/L) in 2009, to 7.8% (>40 U/L) in 2015.^7^, ^8^ In a cross‐sectional study in the southeast of the Kerman province of Iran‐2017, the prevalence of elevated ALT (> 40 U/L in males and >35 U/L in females) was 6.7% in females and 11.7% in males, and it was more prevalent among younger individuals.^9^ In another study conducted in the north‐east of Iran‐2013, the prevalence of elevated ALT (> 40 U/L for both males and females) among individuals aged >50 years was 4% (4.9% in males and 3.5% in females) and more frequent among the younger population.^10^ The difference in the reported prevalence might be related to the different definitions of abnormal ALT.^6^, ^11^, ^12^

In addition to age and gender, abnormal ALT is associated with general and central adiposity and other cardiometabolic components including high blood pressure,^13^, ^14^ high blood glucose, abnormal values of triglycerides (TG), total cholesterol (TC), low‐density lipoprotein cholesterol (LDL‐C), and high‐density lipoprotein cholesterol (HDL‐C).^8^, ^10^, ^15^, ^16^, ^17^ Importantly, few studies found gender‐specific differences in the association between cardiometabolic risk factors and abnormal ALT levels; however, this issue did not address in other studies.^18^, ^19^

Since the current first‐line management of NAFLD rely on increasing physical activity levels, nutritional care, and lifestyle modification, early screening with detection of abnormal ALT could be useful for a variety of noncommunicable diseases such as metabolic syndrome, type‐2 diabetes (T2DM), hypertension, and cardiovascular disease.^20^, ^21^, ^22^, ^23^, ^24^ The level of abnormal ALT differs through populations due to variations in ethnicity, geographic region, and socioeconomic status.^10^, ^15^, ^25^, ^26^ In the current study, we aimed to determine the prevalence of abnormal ALT and its associated components using different criteria among Tehranian males and females in 2018–2022.

## 2 METHODS

### 2.1 Study population

The Tehran Lipids and Glucose Study (TLGS) is a community‐based longitudinal study performed on a Tehranian urban population aged ≥3 years to determine the prevalence and incidence of non‐communicable diseases (NCD) and related risk factors. Volunteer recruitment was done in two different phases, the first (1999–2001; *n* = 15,005) and the second (2001–2005; *n* = 3550), and it is planned to continue for at least 20 years with a tri‐annual interval design (e.g., 3rd phase 2005–2008; 4th phase 2008–2011; 5th phase 2012–2015; 6th phase 2015–2018; and 7th phase 2018–2022). The design and methodology of the TLGS have been reported elsewhere.^27^ In the current study, we included 5676 adults aged 20–70 years who entered the 7th phase (2018–2022) and had data on ALT. After excluding those with cancer (*n* = 34), pregnant women (*n* = 16), and without complete information on other confounders including body mass index (BMI), waist circumference (WC), hypertension, diabetes, TG, HDL‐C, TC, physical activity, smoking, and creatinine (*n* = 308), 5318 individuals (male = 2434) were eligible for the current study (complete case analysis). This study was conducted according to the guidelines laid down in the Declaration of Helsinki, and all procedures involving human subjects were approved by the The Institutional Review Board (IRB) of the Research Institute for Endocrine Sciences (RIES), Shahid Beheshti University of Medical Sciences, Tehran, Iran. Written informed consent was obtained from all subjects.

### 2.2 Clinical and laboratory measurements

Trained interviewers asked all participants about demographics, family history of diabetes (FH‐DM), history of cardiovascular disease (CVD), medication history, education levels, and smoking habits. The subject's anthropometric parameters were measured with light clothing and without shoes. Weight was measured using a digital scale (Seca 707, Seca Corp; range 0.1–150 kg, sensitivity 0.1 kg). Height was measured with a tape meter in a standing position and shoulders in normal alignment. WC at the umbilical was measured by an unscratched tape meter. After 15 min of rest, systolic and diastolic blood pressures (SBP and DBP, respectively) were measured twice on the right arm (with a time interval of 5 min) by a standardized mercury sphygmomanometer (calibrated by the Iranian Institute of Standards and Industrial Researches), and blood pressure was considered a mean of these measurements.

For all subjects after 12–14 h of overnight fasting, a venous blood sample was collected. Fasting plasma glucose (FPG) was measured using an enzymatic colorimetric method with glucose oxidase. TC was assayed using the enzymatic colorimetric method with cholesterol esterase and cholesterol oxidase. Measurement of HDL‐C was done with a homogeneous method (HDL‐C Immuno FS) in which non‐HDL‐C was removed using antibodies against human lipoproteins, and then HDL‐C was measured by the enzymatic colorimetric cholesterol assay. TG was assayed using an enzymatic colorimetric method with glycerol phosphate oxidase. The intra‐ and inter‐assay coefficients of variation (CVs) both were 2.2% for glucose. For both TC and HDL‐Cholesterol, intra‐ and inter‐assay CVs were 0.5% and 2%, respectively. Intra‐ and interassay CVs were 0.6% and 1.6% for TG, respectively. Serum creatinine (SCr) levels were assayed by the kinetic colorimetric Jaffe method. ALT was measured by an optimized UV‐test according to the International Federation of Clinical Chemistry and Laboratory Medicine (IFCC) on a photometry system; their intra‐ and interassay CVs were 2.2% and 3.8% for ALT, respectively. Analysis was performed in the TLGS research laboratory on the same day as blood sampling using commercial kits (Pars Azmoon Inc., Tehran, Iran) and a Selectra 2 auto‐analyzer (Vital Scientific, Spankeren, The Netherlands). To monitor the quality of measurements, lyophilized serum controls in two different normal and pathologically high concentrations (TruLab N and TruLab P; Pars Azmoon Inc.) were used. To assess the repeatability of all assays, in the first run, at least eight repeated measurements were performed and followed by repeated measurements on consequent days. Intra‐assay CV was calculated using data from the first run and interassay CV was calculated using data from all runs. Furthermore, the TLGS laboratory participates in an external quality assurance program. Laboratory analyses of the study samples were performed when both internal and external quality control measures met the acceptance criteria.

### 2.3 Definitions

Age groups were defined as; 20 to <30 years (as reference), 30 to <40 years, 40 to <50 years, 50 to <60 years, and ≥60 years in both genders. BMI was calculated as weight (kg) divided into the square of height (m^2^). BMI was categorized into three groups: 1. <25 kg/m^2^ (as reference); 2. 25–30 kg/m^2^, and ≥30 kg/m^2^. Abdominal obesity was defined as WC ≥95 cm as recommended by The Iranian National Committee of Obesity and based on multiple cross‐sectional and prospective studies.^28^ Marital status is categorized as single (as reference), married, or widowed/divorced. Education was categorized into three groups: 1. <6 years (reference); 2. 6–12 years; and 3. ≥12 years (as reference). Smoking status was defined in three groups; 1. Current smokers as participants who smoked cigarettes daily or occasionally as well as those who used water pipe or pipe; 2. Past smokers as participants who smoked in the past; and 3. Those who never smoked (as reference). A Modifiable Activity Questionnaire (MAQ) was used to collect data on physical activity.^29^, ^30^ We used the average metabolic equivalent of a task (MET) score to define physical activity values and it was categorized as ≥1500, 600–1500, and <600 MET mins/wk (as reference). Positive family history of diabetes was defined as having at least one parent or sibling with diabetes. History of CVD was defined as previous ischemic heart disease and/or cerebrovascular accidents. Diabetes status was defined as follows: normoglycemia (FPG <5.55 mmol/L as reference), prediabetes (FPG: 5.55 to <7 mmol/L), and type 2 diabetes (FPG ≥7 mmol/L or taking diabetes‐lowering medications). Hypertension status was classified as follows: normal (SBP < 120 and DBP < 80 as reference), prehypertension (SBP: 120–140 and DBP: 80–90), and hypertension (SBP ≥140 or DBP ≥90 or taking antihypertensive medications). Heart rate was measured through palpation. High triglycerides were defined as TG ≥6.9 mmol/L. Low HDL‐C was defined as HDL‐C <1.29 mmol/L for females and <1.04 mmol/L for males. Non‐HDL‐C was calculated as TC minus HDL‐C, and it was categorized non‐HDL‐C as <2.59 mmol/L (as reference); 2.59 to <3.37 mmol/L, and ≥3.37 mmol/L.^31^ Pulse rate was classified as, < 60 beats per minute, 60–90 beats per minute (as reference), and ≥90 beats per minute.^32^ Chronic kidney disease (CKD) is defined as either kidney damage or estimated glomerular filtration rate (eGFR) <60 mL/min/1.73 m^2^ for >3 months.^33^ eGFR was estimated from serum creatinine (SCr) values using CKD‐EPI equations^34^: First, SCr values were multiplied by 0.95 before eGFR calculation to standardize SCr^35^, ^36^:

$$\begin{array}{cc} \text{eGFR}=141 & \times \left[\text{the minimum of standardized}\;\mathrm{SCr}\;\left(\mathrm{mg}/\mathrm{dL}\right)/\kappa \;\text{or}\;1\right]\alpha \\ & \times \left[\text{the maximum of standardized}\;\mathrm{SCr}\;\left(\mathrm{mg}/\mathrm{dL}\right)/\kappa \;\text{or}\;1\right]-1.209\\ & \times 0.99\text{3age}\times \left(1.018\;\text{if female}\right)\times \left(1.159\;\text{if black}\right) \end{array}$$

where *κ* is 0.7 for females and 0.9 for males and *α* is −0.329 for females and −0.411 for males.

### 2.4 Definition of abnormal ALT

For defining abnormal serum ALT levels two different thresholds were considered: 1. thresholds recommended by The National Health and Nutrition Examination Survey in the United States (US‐NHANCE) data (ALT ≥30 U/L for females and ≥40 U/L for males)^37^, ^38^ and 2. thresholds recommended in the American College of Gastroenterology (ACG) guideline (ALT >25 U/L for females, and >33 U/L for males).^39^

### 2.5 Statistical analysis

Baseline characteristics of the study population were shown as mean (standard deviation: SD) and number (%) for categorical variables. For covariates with a skewed distribution, the median (interquartile range: IQR) was reported. A comparison of baseline characteristics between normal and abnormal ALT according to two definitions was done by student's *t*‐test for continuous variables, chi‐square test for categorical variables, and Mann–Whitney test for skewed variables. The weighted prevalence of abnormal ALT was calculated using both US‐NHANCE and ACG thresholds. Data were weighted directly to the 2016 urban population of Tehran,^40^ based on the 2016 national Iranian census, to match the age (10‐year strata) and gender strata.

The association of different categorical variables with abnormal ALT (for both US‐NHANCE and ACG definition) was assessed by calculating multivariable‐adjusted odds ratios (ORs) with a 95% confidence interval (CI) using binary logistic regression analysis. A univariable analysis was done for each potential covariate including gender (female as reference), age categories, marital status, education levels, smoking status, physical activity groups, BMI categories, abdominal obesity, glucose tolerance status, blood pressure categories, CKD, high TG, low HDL‐C, non‐HDL‐C categories, lipid‐lowering medications, a steroid medication, FH‐DM, and history CVD. Then, those covariates with a *p*‐value less than 0.2 in the univariable analysis were selected to enter the multivariable model. We evaluated the effect modification of gender and each variable in a multivariable model. All analyses were performed in each gender separately in addition to the total population. All analyses were conducted using STATA version 17 SE (StataCorp, TX, USA), and a two‐tailed *p* < 0.05 was considered significant.

## 3 RESULTS

The study population consists of 5318 participants (male = 2434) with a mean age (SD) of 45.2 years (12.8). The baseline characteristics of participants by ALT levels for both defined thresholds are shown in Table 1. There were significant differences in baseline characteristics between normal and abnormal ALT, except for marital status, education levels, physical activity status, CKD, steroid medication, history of CVD, and FH‐DM. Compared to normal ALT participants in the abnormal group were younger and had higher values for BMI, WC, SBP, DBP, FPG, TG, and non‐HDL‐C; moreover, they also had higher frequencies of smokers and using lipid‐lowering medication.

**TABLE 1:** Baseline characteristics of study population according to different categories of ALT in total population: Tehran lipids and glucose study.

	US‐NHANCE suggested threshold			ACG suggested threshold		
	Normal ALT (*N* = 4658)	Abnormal ALT (*N* = 660)	*p*‐value	Normal ALT (*N* = 4123)	Abnormal ALT (*N* = 1195)	*p*‐value
Age, year	45.5 (12.9)	43.3 (11.8)	<0.001	45.5 (13.0)	44.2 (12.0)	0.003
BMI, kg/m^2^	27.6 (4.9)	29.9 (4.7)	<0.001	27.4 (4.9)	29.5 (4.8)	<0.001
WC, cm	94.6 (11.6)	100.7 (11.1)	<0.001	94.1 (11.7)	99.4 (11.1)	<0.001
SBP, mmHg	111.2 (15.2)	114.7 (13.1)	<0.001	111.0 (15.2)	114.0 (13.8)	<0.001
DBP, mmHg	75.3 (9.4)	78.2 (9.6)	<0.001	75.1 (9.3)	77.7 (9.6)	<0.001
FPG, mmol/L	5.56 (1.61)	5.97 (1.88)	<0.001	5.52 (1.57)	5.92 (1.83)	<0.001
TG, mmol/L	1.36 (0.97–1.93)	1.81 (1.26–2.60)	<0.001	1.32 (0.95–1.89)	1.69 (1.23–2.45)	<0.001
HDL‐C, mmol/L	1.22 (0.29)	1.14 (0.28)	<0.001	1.23 (0.28)	1.16 (0.29)	<0.001
Non‐HDL‐C, mmol/L	3.51 (0.97)	3.86 (1.07)	<0.001	3.48 (0.96)	3.81 (1.06)	<0.001
Heart rate, beats per minute	75.93 (10.0)	76.82 (10.21)	<0.001	75.90 (9.91)	76.58 (10.36)	0.037
Marital status			0.34			0.58
Single	876 (18.1)	136 (20.6)		782 (19.0)	230 (19.2)	
Married	3476 (74.6)	488 (73.9)		3068 (74.4)	896 (75.0)	
Widow/divorced	306 (6.6)	36 (5.4)		273 (6.6)	69 (5.8)	
Education, years			0.18			0.07
<6	314 (6.7)	33 (5.0)		285 (6.9)	62 (5.2)	
6–12	2263 (48.6)	317 (48.0)		2005 (48.6)	572 (48.1)	
≥12	2081 (44.7)	310 (47.0)		1833 (44.5)	558 (46.7)	
Smoking status			<0.001			0.001
Never	2961 (63.6)	350 (53.0)		2624 (63.6)	687 (57.5)	
Past smoker	377 (8.1)	59 (9.0)		324 (7.9)	112 (9.4)	
Current smoker	132 (28.3)	251 (38.0)		1175 (35.8)	396 (33.1)	
Physical activity levels, MET × min/week			0.08			0.59
<600	2105 (45.2)	329 (49.8)		1873 (45.4)	561 (46.9)	
600–1500	1170 (25.1)	154 (23.3)		1028 (24.9)	296 (24.8)	
≥1500	1383 (29.7)	177 (26.8)		1222 (29.6)	338 (28.3)	

BMI categories, kg/m^2^			<0.001			<0.001
<25	1430 (30.7)	90 (13.6)		1318 (31.97)	202 (16.9)	
25–30	1924 (41.3)	253 (38.3)		1698 (41.18)	479 (40.1)	
≥30	1304 (28.0)	317 (48.0)		1107 (26.8)	514 (43.0)	
Central obesity	2275 (48.8)	467 (70.8)	<0.001	1955 (47.4)	787 (65.9)	<0.001
Diabetes status			<0.001			<0.001
Normal	3157 (67.8)	359 (54.4)		2855 (69.2)	661 (55.3)	
Pre‐diabetes	887 (19.0)	167 (25.3)		760 (18.4)	294 (24.6)	
T2DM	614 (13.2)	134 (20.3)		508 (12.3)	240 (20.1)	
Hypertension status			<0.001			<0.001
Normal	2844 (61.1)	331 (50.2)		2550 (61.8)	625 (52.3)	
Pre‐hypertension	1039 (22.3)	183 (27.7)		895 (21.7)	327 (27.4)	
Hypertension	775 (16.6)	146 (22.1)		678 (16.4)	243 (20.3)	
CKD, yes	585 (12.6)	87 (13.2)	0.65	512 (12.4)	160 (13.4)	0.37
High TG	1591 (34.2)	354 (53.6)	<0.001	1344 (32.6)	601 (50.3)	<0.001
Low HDL‐C	2038 (43.7)	337 (51.1)	<0.001	1779 (43.1)	596 (49.9)	<0.001
Non‐HDL‐C categories, mmol/L			<0.001			<0.001
<2.59	781 (16.8)	74 (11.2)		716 (17.4)	139 (11.6)	
2.59–3.37	1387 (29.8)	138 (20.9)		1252 (30.4)	273 (22.8)	
≥3.37	2490 (53.5)	448 (67.9)		2155 (52.3)	783 (65.2)	
Lipid‐lowering medication, yes	697 (15.0)	127 (19.2)	0.004	589 (14.3)	235 (16.7)	<0.001
Steroid medication, yes	99 (2.1)	11 (1.7)	0.44	86 (2.1)	24 (2.0)	0.87
History CVD, yes	309 (6.6)	43 (6.5)	0.91	267 (6.5)	85 (7.1)	0.43
FH‐DM, yes	2103 (45.1)	305 (46.2)	0.61	1851 (44.9)	557 (46.6)	0.29u

*Note*: Data are reported as mean (SD) for normally distributed variables, median (IQR) for skewed, and frequency (%) for categorical variables. NHANCE cut point for abnormal ALT: ALT >40 U/L for males and >31 U/L for females. ACG clinical guideline for abnormal ALT: ALT >33 U/L for males and >25 U/L for females.

Abbreviations: ACG, the American College of Gastroenterology; ALT, alanine aminotransferase; BMI, body mass index; CI, confidence interval; CKD, chronic kidney disease; CVD, cardiovascular disease; DBP, diastolic blood pressure; FH‐DM, family history diabetes; FPG, fasting plasma glucose; HDL‐C, high‐density lipoprotein cholesterol; HDL‐C, high‐density lipoprotein cholesterol; IQR, interquartile range; OR, odds ratio; SBP, systolic blood pressure; SD, standard deviation; T2DM, type 2 diabetes; TG, triglycerides; US‐NHANCE, the National Health and Nutrition Examination Survey in the United States.

**FIGURE 1:**
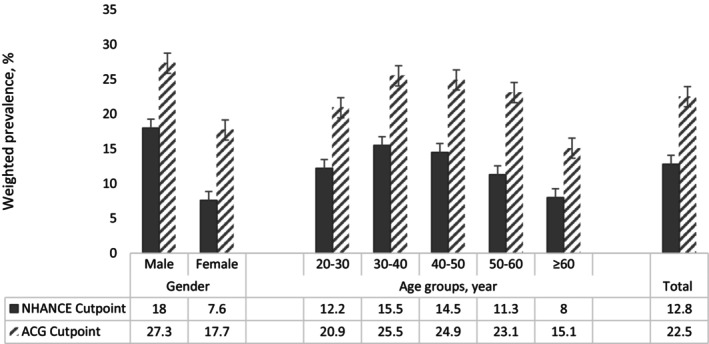
Weighted prevalence of abnormal ALT in different subgroups based on the 7th examination (2018–2022) of TLGS data. ALT, Alanine aminotransferase; TLGS, Tehran lipids and glucose study. US‐NHANCE cut point for abnormal ALT: ALT >40 U/L for male and >31 U/L for female. ACG clinical guideline for abnormal ALT: ALT >33 U/L for male and >25 U/L for female.

In the total population, the weighted mean (95% CI) value of ALT level for individuals aged 20–30 years was significantly higher compared to those ≥60 years [22.9 (21.7–24.0) vs. 21 (20.2–21.8) U/dL, respectively] (Table S1). As shown in Figure 1, the weighted prevalence of abnormal ALT according to ACG guideline was significantly higher compared to US‐NHANCE suggested threshold. Considering US‐NHANCE suggested threshold the weighted prevalence (%) (95% CI) of abnormal ALT was 18.0 (16.6–19.8), 7.6 (6.7–8.7), and 12.8 (11.9–13.8) in males, females, and total population, respectively. Also, individuals with abnormal ALT were more likely to be younger [the weighted prevalence (95% CI): 12.2 (10.0–14.8), 15.5 (13.6–17.5), 14.5 (12.5–16.6), 11.3 (9.6–13.2), 8.0 (6.3–10.0) in subjects 20–30, 30–40, 40–50, 50–60, and ≥ 60 years, respectively]. Considering the ACG guideline, the weighted prevalence (95% CI) of abnormal ALT was 27.3 (25.5–29.2), 17.7 (16.3–19.3), and 22.5 (21.3–23.7) in males, females, and total population, respectively (Table S1). As the same as the US‐NHANCE suggested threshold, the prevalence of abnormal ALT is higher among younger individuals. Since BMI is known to be a significant source of risk for abnormal ALT, as a sensitivity analysis, BMI‐adjusted prevalence was also calculated and the results remained essentially the same (data not shown).

Results of the uni‐ and multivariable logistic regression analysis for abnormal ALT using different definitions are illustrated in Tables 2 and 3, respectively. In the multivariable analysis among the total population for both suggested thresholds, the male gender, BMI ≥25 kg/m^2^ (overweight and obese), non‐HDL‐C ≥3.37 mmol/L, FPG ≥5.55 mmol/L (prediabetes and T2DM), high TG, and lipid‐lowering medications were significantly associated with an increased level of abnormal ALT. Moreover, the results show that a heart rate ≥90 beats per min increased the odds of abnormal ALT (US‐NHANCE criteria) with the odds of 1.37 (1.06–1.76) while central obesity increased the odds of abnormal ALT (ACG criteria).

**TABLE 2:** Univariable and multivariable logistic regression models of predictors of abnormal ALT according to US‐NHANCE suggested threshold among the total population: Tehran lipids and glucose study.

	Univariable		Multivariable	
	OR (95% CI)	*p*‐value	OR (95% CI)	*p*‐value
Gender, male	2.20 (1.86–2.60)	<0.00	**1.98 (1.61–2.43)**	**<0.001**
Age groups, year				
20–30	1.66 (1.19–2.32)	**0.0**	**3.97 (2.51–6.27)**	**<0.001**
30–40	2.06 (1.55–2.74)	<0.001	**3.98 (2.75–5.78)**	**<0.001**
40–50	1.71 (1.28–2.30)	<0.001	**2.66 (1.88–3.76)**	**<0.001**
50–60	1.47 (1.09–1.97)	0.01	**1.63 (1.18–2.27)**	**0.003**
≥60	Reference		Reference	
Marital status				
Single	Reference		Reference	
Married	0.90 (0.74–1.11)	0.33	0.91 (0.70–1.19)	0.51
Widow/divorced	0.76 (0.51–1.12)	0.16	1.02 (0.64–1.60)	0.94
Education, years				
<6	Reference		Reference	
6–12	1.33 (0.91–1.94)	0.13	0.99 (0.65–1.51)	0.98
≥12	1.42 (0.97–2.07)	0.07	1.01 (0.65–1.57)	0.96
Smoking status				
Never	Reference		Reference	
Past smoker	1.32 (0.98–1.78)	0.06	0.89 (0.64–1.22)	0.47
Current smoker	1.61 (1.35–1.91)	<0.001	1.10 (0.90–1.35)	0.36
Physical activity, MET × min/week				
<600	Reference		Reference	
600–1500	0.84 (0.69–1.03)	0.1	0.96 (0.77–1.19)	0.70
≥1500	0.82 (0.67–0.99)	0.04	0.83 (0.67–1.02)	0.07
BMI categories, kg/m^2^				
<25	Reference		Reference	
25–30	2.09 (1.63–2.68)	<0.001	**1.61 (1.21–2.17)**	**0.001**
≥30	3.86 (3.02–4.94)	<0.001	**2.79 (1.97–3.93)**	**<0.001**
Central obesity	2.53 (2.12–3.03)	<0.001	1.29 (0.97–1.63)	0.07
Diabetes status				
Normal	Reference		Reference	
Pre‐diabetes	1.66 (1.36–2.02)	<0.001	**1.45 (1.17–1.80)**	**0.001**
T2DM	1.92 (1.54–2.38)	<0.001	**1.90 (1.46–2.47)**	**<0.001**
Hypertension status				
Normal	Reference		Reference	
Pre‐hypertension	1.51 (1.25–1.84)	<0.00	0.90 (0.54–1.50)	0.69
Hypertension	1.62 (1.31–2.00)	**<0.001**	1.14 (0.89–1.45)	0.29
Heart rate, beats per minute				
60–90	Reference		Reference	
<60	1.11 (0.61–2.00)	0.74	1.23 (0.66–2.27)	0.52
≥90	1.37 (1.06‐1.76)	0.01	**1.35 (1.03–1.77)**	**0.03**
CKD, yes	1.06 (0.83–1.35)	0.65	–	–
High TG	2.23 (1.89–2.63)	<0.001	**1.35 (1.10–1.64)**	**0.003**
Low HDL‐C	1.34 (1.14–1.58)	<0.001	1.07 (0.89–1.29)	0.43
Non‐HDL‐C categories, mmol/L				
<2.59	Reference		Reference	
2.59–3.37	1.05 (0.78–1.41)	0.75	1.00 (0.73–1.37)	0.99
≥3.37	1.90 (1.46–2.46)	<0.001	**1.58 (1.17–2.14)**	**0.003**
Lipid‐lowering medication, yes	1.35 (1.10–1.67)	0.005	**1.83 (1.39–2.45)**	**<0.001**
Steroid medication, yes	0.78 (0.42–1.46)	0.44	–	–
History CVD, yes	0.98 (0.70–1.36)	0.91	–	–
FH‐DM, yes	1.04 (0.89–1.23)	0.61	–	–

*Note*: NHANCE cut point for abnormal ALT: ALT >40 U/L for males and >31 U/L for females.

Significant values of multivariable regression (p‐value <0.05) are bolded.

Abbreviations: ALT, alanine aminotransferase; BMI, body mass index; CI, confidence interval; CKD, chronic kidney disease; CVD, cardiovascular disease; FH‐DM, family history diabetes; HDL‐C, high‐density lipoprotein cholesterol; MET, metabolic equivalent of a task; OR, odds ratio; T2DM, type 2 diabetes; US‐NHANCE, the National Health and Nutrition Examination Survey in the United States.

**TABLE 3:** Univariable and multivariable logistic regression models of predictors of abnormal ALT according to ACG clinical guideline suggested threshold among the total population: Tehran lipids and glucose study.

	Univariable logistic		Multivariable logistic	
	OR (95% CI)	*p*‐value	OR (95% CI)	*p*‐value
Gender, male	1.46 (1.29–1.67)	<0.001	**1.26 (1.08–1.47)**	**0.004**
Age groups, year				
20–30	1.47 (1.14–1.91)	0.003	**3.65 (2.64–5.05)**	**<0.001**
30–40	1.84 (1.48–2.29)	<0.001	**3.55 (2.67–4.72)**	**<0.001**
40–50	1.68 (1.34–2.10)	<0.001	**2.53 (1.93–3.30)**	**<0.001**
50–60	1.66 (1.33–2.08)	<0.001	**1.83 (1.43–2.34)**	**<0.001**
≥60	Reference		Reference	
Marital status				
Single	Reference		Reference	
Married	1.00 (0.84–1.17)	0.93	–	–
Widow/divorced	0.86 (0.63–1.16)	0.33	–	–
Education, years				
<6	Reference		Reference	
6–12	1.32 (0.99–1.76)	0.06	1.13 (0.82–1.56)	0.46
≥12	1.40 (1.05–1.87)	0.023	1.21 (0.86–1.70)	0.26
Smoking status				
Never	Reference		Reference	
Past smoker	1.32 (1.05–1.66)	0.02	1.10 (0.85–1.42)	0.45
Current smoker	1.29 (1.12–1.48)	<0.001	1.06 (0.90–1.26)	0.46
Physical activity, MET × min/week				
<600	Reference		Reference	
600–1500	0.96 (0.82–1.13)	0.63	–	–
≥1500	0.92 (0.79–1.08)	0.31	–	–
BMI categories, kg/m^2^				
<25	Reference		Reference	
25–30	1.84 (1.54–2.20)	<0.001	**1.43 (1.16–1.76)**	**0.001**
≥30	3.03 (2.53–3.63)	<0.001	**2.09 (1.62–2.70)**	**<0.001**
Central obesity	2.14 (1.87–2.45)	<0.001	**1.27 (1.05–1.55)**	**0.01**
Diabetes status				
Normal	Reference		Reference	
Prediabetes	1.67 (1.42–1.96)	<0.001	**1.51 (1.27–1.79)**	**<0.001**
T2DM	2.04 (1.71–2.43)	<0.001	**1.97 (1.60–2.43)**	**<0.001**
Hypertension status				
Normal	Reference		Reference	
Prehypertension	1.49 (1.28–1.74)	<0.001	1.02 (0.67–1.56)	0.91
Hypertension	1.46 (1.23–1.73)	<0.001	1.05 (0.86–1.27)	0.64
Heart rate, beats per minute				
60–90	Reference		Reference	
<60	1.01 (0.62–1.63)	0.97	1.19 (0.72–1.97)	0.49
≥90	1.26 (1.02–1.55)	0.03	1.20 (0.96–1.50)	0.10
CKD, yes	1.09 (0.90–1.32)	0.37	–	–
High TG	2.09 (1.83–2.38)	<0.001	**1.40 (1.20–1.64)**	**<0.001**
Low HDL‐C	1.31 (1.15–1.49)	<0.001	1.04 (0.89–1.20)	0.60
Non‐HDL‐C categories, mmol/L	Reference			
<2.59	1.12 (0.90–1.40)	0.31	Reference	
2.59–3.37	1.87 (1.53–2.28)	<0.001	1.10 (0.87–1.40)	0.41
≥3.37			**1.66 (1.31–2.10)**	**<0.001**
Lipid‐lowering medication, yes	1.47 (1.24–1.74)	<0.001	**1.91 (1.53–2.37)**	**<0.001**
Steroid medication, yes	0.96 (0.61–1.52)	0.87	–	–
History CVD, yes	1.10 (0.86–1.42)	0.43	–	–
FH‐DM, yes	1.07 (0.94–1.22)	0.29	–	–

*Note*: ACG clinical guideline for abnormal ALT: ALT >33 U/L for males and >25 U/L for females.

Significant values of multivariable regression (p‐value <0.05) are bolded.

Abbreviations: ACG, the American College of Gastroenterology; ALT, alanine aminotransferase; BMI, body mass index; CI, confidence interval; CKD, chronic kidney disease; CVD, cardiovascular disease; FH‐DM, family history diabetes; HDL‐C, high‐density lipoprotein cholesterol; MET, metabolic equivalent of a task; OR, odds ratio; T2DM, type 2 diabetes.

Gender‐split multivariable‐adjusted ORs (95% CI) of potential determinants for elevated ALT for different criteria are presented in Tables S2–S5. Among the male, in both defined thresholds, age, BMI ≥25 kg/m^2^, FPG ≥5.55 mmol/L (prediabetes and T2DM), and high TG were significantly associated with an increased level of abnormal ALT, and hypertension shows a significant association using US‐NHANCE threshold [1.50 (1.10–12.03)]. Heart rate ≥ 90 beats per min increased the odds of abnormal ALT using ACG guideline [1.54 (1.11–2.14)] (Tables S2 and S4). Among females, past‐smoker was associated with abnormal ALT using ACG definition [2.21(1.36–3.59)], while other variables including age categories, central obesity, prediabetes/diabetes, non‐HDL‐C ≥3.37 mmol/L, and lipid‐lowering medication were associated with abnormal ALT using both thresholds (Tables S3 and S5).

To show the robustness of our results, a series of sensitivity analyses were conducted. First, a sex‐specific cut point of elevated WC using the national survey of risk factors for noncommunicable diseases of Iran (≥89 m for males and ≥91 cm for females) was considered.^41^ In line with previous findings, considering multivariable analysis among the total population for both suggested thresholds, elevated WC were significantly associated with an increased level of abnormal ALT [1.38 (1.01–1.89) for US‐NHANCE criteria and 1.44 (1.15–1.81)]. Second, a multiple imputation (MI) analysis was also performed for comparison and the result did not change (data not shown).

## 4 DISCUSSION

In a population‐based study of middle‐aged Tehranian residents, the weighted prevalence of abnormal ALT ranged from 12.8% to 22.5% applying US‐NHANCE and ACG criteria, respectively. We also found that generally younger age, male gender, being overweight/obese, central adiposity, TG ≥6.9 mmol/L, non‐HDL‐C ≥3.37 mmol/L, lipid‐lowering medications, and pre‐diabetes/T2DM were associated with abnormal ALT using different definitions. Moreover, among men resting tachycardia, and hypertension, and for females, past‐smoker were also found as another determinant of abnormal ALT.

Our reported prevalence of abnormal ALT in the current study was generally higher than those reported in developed countries, estimated at 11.2% in the Australian population^42^ and 11% in US individuals^43^ using the US‐NAHANCE threshold. However, the prevalence according to ACG guidelines (27.3% in males, 17.7% in females) was higher than the US‐NHANCE suggested threshold (18.1% in males, 7.6% in females). In previous reports in north‐eastern Iran the prevalence of elevated ALT (≥40 U/L for both males and females) among individuals aged over 50 years was 4%; by decreasing the threshold of elevated ALT (>21.4 U/L in males, >18.8 U/L in females) the prevalence of elevated ALT increased to 38%.^10^ In another case–control study conducted in the north of Iran, the prevalence of ALT >40 U/L was about 7.8%.^15^

Several cross‐sectional studies have investigated the association of abnormal ALT with gender and the majority of them showed that male gender is an independent risk factor for ALT elevation. Our results showed that the male gender had higher odds of having elevated ALT according to different thresholds, similar results were found in the Australian and Mexican populations.^42^, ^44^ However, in the study conducted in Germany,^45^ the female gender was significantly correlated with elevated ALT.

With increasing age, the prevalence of abnormal ALT declines significantly among the Tehranian population, an issue that was addressed in previous studies.^9^, ^42^, ^46^ As reported by Mahady et.al,^42^ each decade increase in age decreases the odds of elevated ALT by 29% among Australians, while this was reported to be 18% in the Dutch population.^45^ Our results showed every decade increase in age decreased the risk of abnormal ALT by 32% in the multivariable‐adjusted model. In agreement with previously published studies,^10^, ^44^, ^47^ individuals aged 20–30 years had about four times greater odds of abnormal ALT, while subjects 40–50 years had approximately 2.5 fold grater odds, compared with subjects aged ≥60 years.

The prevalence of abnormal ALT is higher among overweight and obese individuals using both US‐NHANCE and ACG thresholds. We also observed that overweight increase the odds of abnormal ALT by 60% and 40% using US‐NHANCE and ACG threshold, receptively; for obesity, this was found to be more than twofold, similar to those found by Gutiérrez E et.al.^10^, ^44^ We further found that central obesity increases the odds of abnormal ALT by 28% only using the ACG guideline threshold. Hsieh MH et al^48^ reported that in comparison with BMI, WC might be a better indicator of the risk of abnormal liver function among Taiwanese adults. From the data reported by the Taiwanese adults aged 60–64 years, BMI ≥23 kg/m^2^ and central obesity were associated with high ALT [1.54 (1.34–1.76) and 1.74 (1.58–1.93), respectively].^48^ Considering the gender differences in the association of BMI and WC with ALT levels in our study, central obesity was associated with a higher prevalence of abnormal ALT only among women and general obesity in both genders. In the previous study among the Iranian population, Kabir et.al^10^ showed that BMI and WC were associated with a higher level of ALT only among males but not females.

Prediabetes/diabetes was associated with abnormal ALT after adjustment for the BMI, WC, TG, non‐HDL‐C, hypertension, and FH‐DM. Several studies in Iran,^9^, ^49^ Mexico,^44^ and Australia^42^ have investigated the association of ALT levels with type 2 diabetes. In the previous nested case–control study in TLGS, Tohidi et.al^49^ showed that among liver enzymes, only ALT was significantly associated with T2DM even after adjustment with classic risk factors[3.18 (1.02–9.86)]. In another study, a dose–response increase was reported for ALT and T2DM.^9^ However, in our study, both genders showed a significant association between high FPG and abnormal ALT, but most of the studies reported this association only among women.^44^, ^50^, ^51^ A sex‐specific association from the cardiorespiratory fitness study in armed forces (CHIEF) in eastern Taiwan^52^ suggested that FPG ≥5.55 mmol/L in women is highly correlated with ALT ≥40 U/L after adjustment with metabolic risk factors [7.59 (2.35–24.51)].

Considering lipid profiles, TG ≥6.9 mmol/L, non‐HDL‐C ≥3.37 mmol/L, and lipid‐lowering medications were independently associated with abnormal ALT in the total population. A gender‐specific analysis shows that high triglyceridemia was associated with abnormal ALT only among males, while the last two variables were associated only with females. In previous Iranian studies, hypertriglyceridemia was associated with elevated ALT in both genders.^10^, ^15^

The influence of hypertension on abnormal ALT was observed only among men using the US‐NHANCE threshold. This positive association among men was reported by the CHIEF Study^52^ in the Taiwanese population [1.40 (1.19–1.65)] and rural Chinese population^46^ [1.33 (1.08–1.62)]. Concerning previous studies, current smoking showed a negative association with elevated ALT,^45^ especially among males,^46^ past smokers increased the odds of abnormal ALT (ACG guideline threshold) only among females.

Our study showed positive associations between abnormal serum ALT levels and heart rate ≥90 beats per minute in the total population using US‐NHANCE threshold and among males in ACG guideline definition even after multiple adjustments with metabolic risk factors as well as physical activity levels that did not remain as an independent factor. This significant association was reported by Laine et.al^53^ among overweight/obese Finish adults. In another study, Straznicky et al.^54^ reported that ALT was positively associated with resting HR in obese subjects with metabolic syndrome. Moreover, Kim et al.^55^ reported a positive association between resting HR and NAFLD among post‐menopausal women. In our analysis, this association remained a significant event after adjustment with central and general obesity, hypertension, diabetes, and lipid profiles, which may be a representative marker for heightened stress levels and autonomic nervous system imbalance.^53^ Therefore, measuring heart rate as another marker for evaluation of abnormal ALT is recommended, specifically among men.

Several limitations in the current study need to be acknowledged. First, since it was a cross‐sectional study we were not able to determine the causal effect of metabolic factors on abnormal ALT. Second, we did not measure markers of hepatitis B and C infectious as well as other liver enzymes such as γ‐glutamyltranspeptidase or common medications that may cause elevated ALT including antibiotics, antiepileptics, nonsteroidal anti‐inflammatory agents, anti‐tuberculosis drugs, anti‐retroviral treatment for HIV, biologic agents such as anti‐tumor necrosis factor drugs, and some cancer chemotherapeutic agents. Third, despite widespread adjustment in our study, unmeasured confounders such as alcohol consumption may explain part of this association. Alcohol consumption is a great risk factor for liver damage and it's estimated that about 35% of alcohol drinkers developed early liver disease; the issue that might affected by the individual gut microbiome.^56^, ^57^ According to the systematic review and meta‐analysis study,^58^ one out of eight general population of Iran reported consumption of alcohol despite its legal prohibition. Therefore further study is required to evaluate alcohol‐related harms for liver disease in Iran. Although the number of abnormal ALT might be relatively small, our analysis shows the statistical power was enough to guarantee statistical significance with the OR of 1.27 or greater (power = 95%). However, the power for detecting the OR of 1.21 (e.g education levels) was not enough (power = 50%). However, the population‐based study, measured values of metabolic risk factors, and relevant laboratory data are some of the strengths of the current study.

## 5 CONCLUSIONS

In conclusion, we found that out of every 100‐person, about 18–27 males and 8–18 females have abnormal ALT. We also observed male sex and younger age showed significant risk factors for abnormal ALT among the Tehranian population. Obesity, prediabetes/T2DM, hypertriglyceridemia, high non‐HDL‐C in both genders, as well as resting tachycardia and hypertension for men and past‐smoker for women had the most consistent association with abnormal ALT. Hence, the high prevalence of abnormal ALT among non‐elderly Iranian adults, especially among men, necessitates immediate multifaceted strategies by policymakers to prevent potential complications caused by NAFLD.

## AUTHOR CONTRIBUTIONS

SA, FH conceived and planned the study. A.AM and FA cooperated in data gathering. SA conducted the analyses. SA, MT, DM, and FH, developed the first draft of the manuscript. All authors have read and approved the final manuscript.

## FUNDING INFORMATION

This study was supported by the Shahid Beheshti University of Medical Sciences, Tehran, Iran [grant no. 02‐43004160].

## CONFLICT OF INTEREST STATEMENT

None declared.

## Supporting information


Table S1

## Data Availability

The datasets used and/or analyzed during the current study are available from the corresponding author upon reasonable request.
