# Supplementary material for: Prevalence and metabolic determinants of abnormal alanine aminotransferase: A cross‐sectional study of Iranian adults, 2018–2022
Source: J Clin Lab Anal. 2023 Jul 5;37(11-12):e24937. doi: 10.1002/jcla.24937 (PMC10431421; doi:10.1002/jcla.24937)
Supplement: Supplementary file 1 — Table S1 [file JCLA-37-e24937-s001.docx]

| **Supplementary Table 1: Weighted mean (95 % CI) ALT levels and prevalence (95 % CI) of abnormal ALT in different subgroups based on the 7th examination (2018-2022) of TLGS data.** | | | | | | |
| --- | --- | --- | --- | --- | --- | --- |
|  |  | **US-NHANCE cut point** | |  | **ACG clinical guideline** | |
|  | **Mean (95% CI)** | **n/N** | **Prevalence (95 % CI)** |  | **n/N** | **Prevalence (95 % CI)** |
| **Male** | 29.0(28.3-129.8) | 414/2434 | 18.01(16.6-19.8) |  | 635/2434 | 27.3(25.5-29.2) |
| **Age groups, years** |  |  |  |  |  |  |
| - **20-30** | 29.6(27.6-31.6) | 68/349 | 19.5(15.6-24.0) |  | 98/349 | 28.1(23.6-33.0) |
| - **30-40** | 32.6(31.1-34.2) | 160/648 | 24.7(21.5-28.2) |  | 236/648 | 36.4(32.8-40.2) |
| - **40-50** | 30.8(29.3-32.4) | 106/500 | 21.2(17.8-25.0) |  | 155/500 | 31.0(27.1-35.2) |
| - **50-60** | 25.2(24.2-26.2) | 54/555 | 9.7(7.5-12.5) |  | 102/555 | 18.4(15.4-21.8) |
| - **≥60** | 22.3(21.1-23.4) | 26/382 | 6.8(4.7-9.8) |  | 44/382 | 11.5(6.7-15.1) |
|  |  |  |  |  |  |  |
| **Female** | 18.1(17.7-18.6) | 246/2884 | 7.6(6.7-8.7) |  | 560/2884 | 17.7(16.3-19.3) |
| **Age groups, years** |  |  |  |  |  |  |
| - **20-30** | 16.4(15.2-17.6) | 16/308 | 5.2(3.2-8.3) |  | 43/308 | 14.0(10.5-18.3) |
| - **30-40** | 17.4(16.5-18.3) | 42/664 | 6.3(4.7-8.5) |  | 98/664 | 14.7(12.2-17.7) |
| - **40-50** | 17.9(17.2-18.6) | 54/719 | 7.5(5.8-9.7) |  | 134/719 | 18.6(15.9-21.6) |
| - **50-60** | 21.1(20.3-22.0) | 88/686 | 12.8(10.5-15.5) |  | 190/686 | 27.7(24.5-31.1) |
| - **≥60** | 19.8(18.6-20.9) | 46/507 | 9.1(6.8-11.9) |  | 95/507 | 18.7(15.6-22.4) |
|  |  |  |  |  |  |  |
| **Total** | 23.6(23.1-24.0) | 660/5318 | 12.8(11.9-13.8) |  | 1195/5318 | 22.5(21.3-23.7) |
| **Age groups, years** |  |  |  |  |  |  |
| - **20-30** | 22.9(21.7-24.0) | 84/657 | 12.2(10.0-14.8) |  | 141/657 | 20.9(18.0-24.1) |
| - **30-40** | 25.0(24.1-25.8) | 202/1312 | 15.5(13.6-17.5) |  | 334/1312 | 25.5(23.3-27.9) |
| - **40-50** | 24.5(23.6-25.3) | 160/1219 | 14.5(12.5-16.6) |  | 289/1219 | 24.9(22.5-27.5) |
| - **50-60** | 23.1(22.5-23.8) | 142/1241 | 11.3(9.6-13.2) |  | 292/1241 | 23.1(20.8-25.5) |
| - **≥60** | 21.0(20.2-21.8) | 72/889 | 8.0(6.3-10.0) |  | 139/889 | 15.1(12.9-17.6) |
| ALT: Alanine aminotransferase; TLGS: Tehran lipids and glucose study.  NHANCE cut point for abnormal ALT: ALT >40 U/L for males and >31 U/L for females  ACG clinical guideline for abnormal ALT: ALT >33 U/L for males and >25 U/L for females | | | | | | |

| **Supplementary Table 2: Univariable and multivariable logistic regression models of predictors of abnormal ALT according to US-NHANCE cut point among male: Tehran lipids and glucose study** | | | | |
| --- | --- | --- | --- | --- |
|  | **Univariable** | | **Multivariable** | |
|  | **OR (95 % CI)** | **p-value** | **OR (95 % CI)** | **p-value** |
| **Age groups, year** |  |  |  |  |
| - **20-30** | 3.31(2.05-5.34) | <0.001 | **6.42(3.46-11.95)** | **<0.001** |
| - **30-40** | 4.49(2.90-6.95) | <0.001 | **6.55(3.85-11.15)** | **<0.001** |
| - **40-50** | 3.68(2.34-5.79) | <0.001 | **4.38(2.63-7.28)** | **<0.001** |
| - **50-60** | 1.47(0.91-2.40) | 0.11 | 1.53(0.91-2.57) | 0.11 |
| - **≥60** | Reference |  | Reference |  |
| **Marital status** |  |  |  |  |
| - **Single** | Reference |  | Reference |  |
| - **Married** | 0.78(0.61-0.99) | 0.039 | 0.98(0.71-1.35) | 0.89 |
| - **Widow/divorced** | 0.57(0.25-1.30) | 0.18 | 0.89(0.37-2.16) | 0.80 |
| **Education, years** |  |  |  |  |
| - **<6** | Reference |  | Reference |  |
| - **6-12** | 1.31(0.66-2.59) | 0.43 | 0.64(0.30-1.35) | 0.24 |
| - **≥12** | 1.69(0.86-3.32) | 0.13 | 0.68(0.32-1.45) | 0.31 |
| **Smoking status** |  |  |  |  |
| - **Never** | Reference |  | Reference |  |
| - **Past smoker** | 0.93(0.66-1.31) | 0.67 | 0.90(0.62-1.30) | 0.56 |
| - **Current smoker** | 1.31(1.04-1.65) | 0.02 | 1.09(0.85-1.41) | 0.48 |
| **Physical activity, MET × min/week** |  |  |  |  |
| - **<600** | Reference |  | Reference |  |
| - **600-1500** | 0.93(0.69-1.24) | 0.61 | - | - |
| - **≥1500** | 0.90(0.70-1.14) | 0.38 | - | - |
| **BMI categories, kg/m^2^** |  |  |  |  |
| - **<25** | Reference |  | Reference |  |
| - **25-30** | 1.70(1.25-2.31) | 0.001 | **1.46(1.00-2.14)** | **0.05** |
| - **≥30** | 3.77(2.76-5.16) | <0.001 | **2.82(1.80-4.46)** | **<0.001** |
| **Central obesity** | 2.09(1.66-2.63) | <0.001 | 1.07(0.76-1.50) | 0.68 |
| **Diabetes status** |  |  |  |  |
| - **Normal** | Reference |  | Reference |  |
| - **Pre-diabetes** | 1.29(1.01-1.65) | 0.04 | **1.45(1.10-1.91)** | **0.008** |
| - **T2DM** | 1.28(0.93-1.75) | 0.13 | **2.01(1.36-2.96)** | **<0.001** |
| **Hypertension status** |  |  |  |  |
| - **Normal** | Reference |  | Reference |  |
| - **Pre-hypertension** | 1.27(1.0-1.61) | 0.05 | 1.11(0.62-2.0) | 0.73 |
| - **Hypertension** | 1.42(1.07-1.87) | 0.01 | **1.50(1.10-2.03)** | **0.009** |
| **Heart rate, beats per minute** |  |  |  |  |
| - **60-90** | Reference |  | Reference |  |
| - **<60** | 0.88(0.46-1.69) | 0.70 | 1.40(0.70-2.75) | 0.35 |
| - **≥90** | 1.50(1.06-2.12) | 0.02 | 1.43(0.98-2.08) | 0.06 |
| **CKD, yes** | 0.79(0.53-1.19) | 0.27 | - | - |
| **High Triglycerides** | 2.11(1.70-2.62) | <0.001 | **1.51(1.16-1.96)** | **0.002** |
| **Low HDL-C** | 1.50(1.22-1.86) | <0.001 | 1.13(0.89-1.44) | 0.30 |
| **Non-HDL-C categories, mmol/L** |  |  |  |  |
| - **<2.59** | Reference |  | Reference |  |
| - **2.59-3.37** | 0.98(0.66-1.45) | 0.92 | 0.75(0.49-1.14) | 0.17 |
| - **≥3.37** | 1.91(1.36-2.68) | <0.001 | 1.16(0.79-1.73) | 0.44 |
| **Lipid-lowering medication, yes** | 0.97(0.71-1.32) | 0.86 | - | - |
| **Steroid medication, yes** | 0.75(0.34-1.68) | 0.49 | - | - |
| **History CVD, yes** | 0.81(0.54-1.22) | 0.32 | - | - |
| **FHDM, yes** | 1.08(0.87-1.34) | 0.47 | - | - |
| BMI: body mass index; T2DM: type 2 diabetes; CKD: chronic kidney disease; HDL-C: high density lipoprotein cholesterol; CVD: cardiovascular disease; FHDM: family history diabetes; OR: odds ratio; CI: confidence interval  NHANCE cut point for abnormal ALT: ALT >40 U/L for males and >31 U/L for females | | | | |

| **Supplementary Table 3: Univariable and multivariable logistic regression models of predictors of abnormal ALT according to US-NHANCE cut point among female: Tehran lipids and glucose study** | | | | |
| --- | --- | --- | --- | --- |
|  | **Univariable** | | **Multivariable** | |
|  | **OR (95 % CI)** | **p-value** | **OR (95 % CI)** | **p-value** |
| **Age groups, year** |  |  |  |  |
| - **20-30** | 0.55(0.30-0.99) | 0.046 | **2.10(0.98-4.46)** | **0.05** |
| - **30-40** | 0.66(0.44-1.05) | 0.08 | **1.90(1.09-3.31)** | **0.02** |
| - **40-50** | 0.81(0.54-1.23) | 0.33 | **1.54(0.94-2.57)** | **0.09** |
| - **50-60** | 1.47(1.01-2.15) | 0.04 | **1.82(1.20-2.74)** | **0.004** |
| - **≥60** | Reference |  | Reference |  |
| **Marital status** |  |  |  |  |
| - **Single** | Reference |  | Reference |  |
| - **Married** | 1.96(1.24-3.12) | 0.004 | 1.21(0.70-2.08) | 0.50 |
| - **Widow/divorced** | 2.22(1.24-3.98) | 0.007 | 1.20(0.61-2.35) | 0.60 |
| **Education, years** |  |  |  |  |
| - **<6** | Reference |  | Reference |  |
| - **6-12** | 1.11(0.70-1.77) | 0.64 | - | - |
| - **≥12** | 0.83(0.51-1.34) | 0.44 | - | - |
| **Smoking status** |  |  |  |  |
| - **Never** | Reference |  | Reference |  |
| - **Past smoker** | 1.20(0.592.43) | 0.61 | - | - |
| - **Current smoker** | 0.93(0.65-1.35) | 0.71 | - | - |
| **Physical activity, MET × min/week** |  |  |  |  |
| - **<600** | Reference |  | Reference |  |
| - **600-1500** | 0.96(0.71-1.29) | 0.77 | 0.95(0.70-1.30) | 0.77 |
| - **≥1500** | 0.67(0.48-0.94) | 0.02 | 0.72(0.51-1.03) | 0.07 |
| **BMI categories, kg/m^2^** |  |  |  |  |
| - **<25** | Reference |  | Reference |  |
| - **25-30** | 2.64(1.70-4.08) | <0.001 | **1.85(1.14-3.01)** | **0.01** |
| - **≥30** | 4.69(3.09-7.14) | <0.001 | **2.40(1.38-4.19)** | **0.002** |
| **Central obesity** | 2.90(2.19-3.86) | <0.001 | **1.49(1.01-2.22)** | **0.05** |
| **Diabetes status** |  |  |  |  |
| - **Normal** | Reference |  | Reference |  |
| - **Pre-diabetes** | 2.08(1.49-2.92) | <0.001 | **1.55(1.09-2.22)** | **0.02** |
| - **T2DM** | 3.27(2.39-4.46) | <0.001 | **2.20(1.54-3.16)** | **<0.001** |
| **Hypertension status** |  |  |  |  |
| - **Normal** | Reference |  | Reference |  |
| - **Pre-hypertension** | 1.29(0.91-1.84) | 0.15 | 0.33(0.08-1.42) | 0.14 |
| - **Hypertension** | 1.58(1.13-2.11) | 0.007 | 0.77(0.53-1.13) | 0.19 |
| **Heart rate, beats per minute** |  |  |  |  |
| - **60-90** | Reference |  | Reference |  |
| - **<60** | 0.85(0.19-3.74) | 0.83 | 0.82(0.19-3.63) | 0.80 |
| - **≥90** | 1.42(0.95-2.12) | 0.09 | 1.42(0.95-2.19) | 0.08 |
| **CKD, yes** | 1.68(1.22-2.29) | 0.001 | 1.15(0.80-1.66) | 0.45 |
| **High Triglycerides** | 1.90(1.46-2.48) | <0.001 | 1.06(0.77-1.46) | 0.71 |
| **Low HDL-C** | 1.33(1.02-1.73) | 0.034 | 1.08(0.81-1.45) | 0.57 |
| **Non-HDL-C categories, mmol/L** |  |  |  |  |
| - **<2.59** | Reference |  | Reference |  |
| - **2.59-3.37** | 1.17(0.74-1.85) | 0.50 | 1.24(0.77-2.00) | 0.38 |
| - **≥3.37** | 1.77(1.17-2.67) | 0.006 | **1.76(1.09-2.83)** | **0.02** |
| **Lipid-lowering medication, yes** | 2.15(1.60-2.89) | <0.001 | **1.81(1.22-2.66)** | **0.003** |
| **Steroid medication, yes** | 0.79(0.28-2.20) | 0.65 | - | - |
| **History CVD, yes** | 1.09(0.62-1.92) | 0.76 | - | - |
| **FHDM, yes** | 1.16(0.89-1.51) | 0.25 | - | - |
| BMI: body mass index; T2DM: type 2 diabetes; CKD: chronic kidney disease; HDL-C: high density lipoprotein cholesterol; CVD: cardiovascular disease; FHDM: family history diabetes; OR: odds ratio; CI: confidence interval  NHANCE cut point for abnormal ALT: ALT >40 U/L for males and >31 U/L for females | | | | |

| **Supplementary Table 4: Univariable and multivariable logistic regression models of predictors of abnormal ALT according to ACG clinical guideline among male: Tehran lipids and glucose study** | | | | |
| --- | --- | --- | --- | --- |
|  | **Univariable logistic** | | **Multivariable logistic** | |
|  | **OR (95 % CI)** | **p-value** | **OR (95 % CI)** | **p-value** |
| **Age groups, year** |  |  |  |  |
| - **20-30** | 3.00(2.03-4.35) | <0.001 | **5.99(3.53-10.15)** | **<0.001** |
| - **30-40** | 4.40(3.10-6.26) | <0.001 | **6.76(4.34-10.54)** | **<0.001** |
| - **40-50** | 3.45(2.39-5.00) | <0.001 | **4.24(2.77-6.49)** | **<0.001** |
| - **50-60** | 1.73(1.18-2.53) | <0.001 | **1.81(1.20-2.74)** | **0.005** |
| - **≥60** | Reference |  | Reference |  |
| **Marital status** |  |  |  |  |
| - **Single** | Reference |  | Reference |  |
| - **Married** | 0.81(0.66-0.99) | 0.049 | 0.97(0.73-1.30) | 0.86 |
| - **Widow/divorced** | 0.46(0.22-0.96) | 0.038 | 0.64(0.29-1.43) | 0.28 |
| **Education, years** |  |  |  |  |
| - **<6** | Reference |  | Reference |  |
| - **6-12** | 1.57(0.87-2.83) | 0.13 | 0.83(0.43-1.60) | 0.58 |
| - **≥12** | 1.93(1.07-3.48) | 0.028 | 0.83(0.43-1.61) | 0.59 |
| **Smoking status** |  |  |  |  |
| - **Never** | Reference |  | Reference |  |
| - **Past smoker** | 0.97(0.73-1.29) | 0.83 | - | - |
| - **Current smoker** | 1.17(0.96-1.42) | 0.12 | - | - |
| **Physical activity, MET × min/week** |  |  |  |  |
| - **<600** | Reference |  | Reference |  |
| - **600-1500** | 0.84(0.65-1.08) | 0.18 | 0.80(0.61-1.05) | 0.11 |
| - **≥1500** | 0.95(0.78-1.17) | 0.65 | 0.91(0.73-1.13) | 0.41 |
| **BMI categories, kg/m^2^** |  |  |  |  |
| - **<25** | Reference |  | Reference |  |
| - **25-30** | 1.85(1.44-2.39) | <0.001 | **1.57(1.15-2.15)** | **0.004** |
| - **≥30** | 3.60(2.77-4.69) | <0.001 | **2.68(1.83-3.92)** | **<0.001** |
| **Central obesity** | 2.04(1.68-2.48) | <0.001 | 1.12(0.84-1.48) | 0.45 |
| **Diabetes status** |  |  |  |  |
| - **Normal** | Reference |  | Reference |  |
| - **Pre-diabetes** | 1.38(1.121.71) | 0.003 | **1.57(1.23-1.90)** | **<0.001** |
| - **T2DM** | 1.25(0.95-1.65) | 0.11 | **1.99(1.42-2.79)** | **<0.001** |
| **Hypertension status** |  |  |  |  |
| - **Normal** | Reference |  | Reference |  |
| - **Pre-hypertension** | 1.27(1.03-1.56) | 0.02 | 1.07(0.64-1.80) | 0.76 |
| - **Hypertension** | 1.19(0.93-1.51) | 0.17 | 1.17(0.90-1.54) | 0.24 |
| **Heart rate, beats per minute** |  |  |  |  |
| - **60-90** | Reference |  | Reference |  |
| - **<60** | 0.81(0.46-1.42) | 0.46 | 1.27(0.70-2.31) | 0.39 |
| - **≥90** | 1.57(1.15-2.13) | 0.004 | **1.54(1.11-2.14)** | **0.01** |
| **CKD, yes** | 0.76(0.54-1.08) | 0.13 | 1.35(0.90-2.01) | 0.14 |
| **High Triglycerides** | 2.05(1.71-2.47) | <0.001 | **1.49(1.19-1.87)** | **<0.001** |
| **Low HDL-C** | 1.50(1.25-1.80) | <0.001 | 1.16(0.94-1.43) | 0.15 |
| **Non-HDL-C categories, mmol/L** |  |  |  |  |
| - **<2.59** | Reference |  | Reference |  |
| - **2.59-3.37** | 1.21(0.87-1.67) | 0.25 | 0.93(0.66-1.32) | 0.69 |
| - **≥3.37** | 2.03(1.52-2.70) | <0.001 | 1.22(0.88-1.71) | 0.23 |
| **Lipid-lowering medication, yes** | 0.98(0.75-1.27) | 0.87 | - | - |
| **Steroid medication, yes** | 0.85(0.44-1.62) | 0.62 | - | - |
| **History CVD, yes** | 0.91(0.65-1.28) | 0.59 | - | - |
| **FHDM, yes** | 1.00(0.82-1.19) | 0.94 | - | - |
| BMI: body mass index; T2DM: type 2 diabetes; CKD: chronic kidney disease; HDL-C: high density lipoprotein cholesterol; CVD: cardiovascular disease; FHDM: family history diabetes; OR: odds ratio; CI: confidence interval  ACG clinical guideline for abnormal ALT: ALT >33 U/L for males and >25 U/L for females | | | | |

| **Supplementary Table 5: Univariable and multivariable logistic regression models of predictors of abnormal ALT according to ACG clinical guideline among female: Tehran lipids and glucose study** | | | | |
| --- | --- | --- | --- | --- |
|  | **Univariable logistic** | | **Multivariable logistic** | |
|  | **OR (95 % CI)** | **p-value** | **OR (95 % CI)** | **p-value** |
| **Age groups, year** |  |  |  |  |
| - **20-30** | 0.70(0.48-1.04) | 0.08 | **2.13(1.24-3.63)** | **0.006** |
| - **30-40** | 0.75(0.55-1.02) | 0.07 | **1.86(1.23-2.81)** | **0.003** |
| - **40-50** | 1.00(0.74-1.33) | 0.96 | **1.86(1.29-2.68)** | **0.001** |
| - **50-60** | 1.66(1.26-2.19) | <0.001 | **2.08(1.52-2.84)** | **<0.001** |
| - **≥60** | Reference |  | Reference |  |
| **Marital status** |  |  |  |  |
| - **Single** | Reference |  | Reference |  |
| - **Married** | 1.60(1.19-2.14) | 0.002 | 1.11(0.78-1.59) | 0.55 |
| - **Widow/divorced** | 1.66(1.12-2.46) | 0.012 | 1.03(0.65-1.64) | 0.89 |
| **Education, years** |  |  |  |  |
| - **<6** | Reference |  | Reference |  |
| - **6-12** | 1.16(0.83-1.63) | 0.38 | - | - |
| - **≥12** | 1.03(0.73-1.45) | 0.88 | - | - |
| **Smoking status** |  |  |  |  |
| - **Never** | Reference |  | Reference |  |
| - **Past smoker** | 1.95(1.23-3.08) | 0.004 | **2.21(1.36-3.59)** | **0.001** |
| - **Current smoker** | 0.99(0.76-1.28) | 0.94 | 1.20(0.91-1.59) | 0.19 |
| **Physical activity, MET × min/week** |  |  |  |  |
| - **<600** | Reference |  | Reference |  |
| - **600-1500** | 1.16(0.94-1.44) | 0.17 | 1.19(0.95-1.49) | 0.13 |
| - **≥1500** | 0.88(0.70-1.11) | 0.28 | 0.93(0.72-1.19) | 0.59 |
| **BMI categories, kg/m^2^** |  |  |  |  |
| - **<25** | Reference |  | Reference |  |
| - **25-30** | 1.74(1.35-2.26) | <0.001 | 1.20(0.89-1.61) | 0.22 |
| - **≥30** | 2.69(2.09-3.45) | <0.001 | 1.40(0.97-2.00) | 0.07 |
| **Central obesity** | 2.11(1.75-2.56) | <0.001 | **1.34(1.01-1.77)** | **0.04** |
| **Diabetes status** |  |  |  |  |
| - **Normal** | Reference |  | Reference |  |
| - **Pre-diabetes** | 1.91(1.50-2.44) | <0.001 | **1.50(1.16-1.94)** | **0.002** |
| - **T2DM** | 3.08(2.44-3.89) | <0.001 | **2.32(1.77-3.05)** | **<0.001** |
| **Hypertension status** |  |  |  |  |
| - **Normal** | Reference |  | Reference |  |
| - **Pre-hypertension** | 1.52(1.19-1.93) | 0.001 | 0.78(0.35-1.73) | 0.54 |
| - **Hypertension** | 1.66(1.31-2.12) | <0.001 | 0.92(0.69-1.23) | 0.60 |
| **Heart rate, beats per minute** |  |  |  |  |
| - **60-90** | Reference |  | Reference |  |
| - **<60** | 1.33(0.53-3.34) | 0.55 | - | - |
| - **≥90** | 1.10(0.83-1.47) | 0.51 | - | - |
| **CKD, yes** | 1.46(1.16-1.85) | 0.001 | 1.04(0.79-1.36) | 0.80 |
| **High Triglycerides** | 1.96(1.62-2.37) | <0.001 | 1.24(0.99-1.57) | 0.06 |
| **Low HDL-C** | 1.22(1.01-1.46) | 0.036 | 1.02(0.83-1.25) | 0.83 |
| **Non-HDL-C categories, mmol/L** |  |  |  |  |
| - **<2.59** | Reference |  | Reference |  |
| - **2.59-3.37** | 1.05(0.77-1.44) | 0.73 | 1.12(0.80-1.55) | 0.51 |
| - **≥3.37** | 1.69(1.28-2.23) | <0.001 | **1.72(1.24-3.87)** | **0.001** |
| **Lipid-lowering medication, yes** | 2.11(1.69-2.63) | <0.001 | **1.89(1.41-2.53)** | **<0.001** |
| **Steroid medication, yes** | 1.08(0.57-2.06) | 0.80 | - | - |
| **History CVD, yes** | 1.31(0.89-1.92) | 0.17 | 1.05(0.81-1.20) | 0.89 |
| **FH-DM, yes** | 1.23(1.03-1.49) | 0.02 | 0.99(0.81-1.20) | 0.90 |
| BMI: body mass index; T2DM: type 2 diabetes; CKD: chronic kidney disease; HDL-C: high density lipoprotein cholesterol; CVD: cardiovascular disease; FHDM: family history diabetes; OR: odds ratio; CI: confidence interval  ACG clinical guideline for abnormal ALT: ALT >33 U/L for males and >25 U/L for females | | | | |
